# Polymer of Intrinsic Microporosity (PIM-1) Membranes Treated with Supercritical CO_2_

**DOI:** 10.3390/membranes9030041

**Published:** 2019-03-18

**Authors:** Colin A. Scholes, Shinji Kanehashi

**Affiliations:** 1 Department of Chemical Engineering, The University of Melbourne, VIC 3010, Australia; 2 Graduate School of Engineering, Tokyo University of Agriculture and Technology, Tokyo 184-8588, Japan; kanehasi@cc.tuat.ac.jp

**Keywords:** polymer of intrinsic microporosity, supercritical carbon dioxide, aging, permeability

## Abstract

Polymers of intrinsic microporosity (PIMs) are a promising membrane material for gas separation, because of their high free volume and micro-cavity size distribution. This is countered by PIMs-based membranes being highly susceptible to physical aging, which dramatically reduces their permselectivity over extended periods of time. Supercritical carbon dioxide is known to plasticize and partially solubilise polymers, altering the underlying membrane morphology, and hence impacting the gas separation properties. This investigation reports on the change in PIM-1 membranes after being exposed to supercritical CO_2_ for two- and eight-hour intervals, followed by two depressurization protocols, a rapid depressurization and a slow depressurization. The exposure times enables the impact contact time with supercritical CO_2_ has on the membrane morphology to be investigated, as well as the subsequent depressurization event. The density of the post supercritical CO_2_ exposed membranes, irrespective of exposure time and depressurization, were greater than the untreated membrane. This indicated that supercritical CO_2_ had solubilised the polymer chain, enabling PIM-1 to rearrange and contract the free volume micro-cavities present. As a consequence, the permeabilities of He, CH_4_, O_2_ and CO_2_ were all reduced for the supercritical CO_2_-treated membranes compared to the original membrane, while N_2_ permeability remained unchanged. Importantly, the physical aging properties of the supercritical CO_2_-treated membranes altered, with only minor reductions in N_2_, CH_4_ and O_2_ permeabilities observed over extended periods of time. In contrast, He and CO_2_ permeabilities experienced similar physical aging in the supercritical treated membranes to that of the original membrane. This was interpreted as the supercritical CO_2_ treatment enabling micro-cavity contraction to favour the smaller CO_2_ molecule, due to size exclusion of the larger N_2_, CH_4_ and O_2_ molecules. Therefore, physical aging of the treated membranes only had minor impact on N_2_, CH_4_ and O_2_ permeability; while the smaller He and CO_2_ gases experience greater permeability loss. This result implies that supercritical CO_2_ exposure has potential to limit physical aging performance loss in PIM-1 based membranes for O_2_/N_2_ separation.

## 1. Introduction

Polymers of intrinsic microporosity (PIMs) are attractive for polymeric membranes, because of their very high fractional free volume and favourable interconnectivity between micro-cavities [1,2]. For many gas pairs, PIMs-based membranes are on or above the Robeson’s upper bound, the criterion denoting current state-of-art performance in gas separation membranes [3]. This high performance is the result of the spirobisindane moiety, creating rigid ladder-type polymeric chain structures, with significant steric hindrance preventing chain rotation and limiting chain packing. However, PIMs-based polymeric membranes suffer from a reduction in separation performance over time, known as physical aging [4]. This is evident by an initial rapid decline in gas permeability on the order of days, which tampers off to a gradual decline over extended time periods [5]. This physical aging phenomenon in PIMs is similar to behaviour reported for other very high fractional free volume polymers, such as poly [1-(trimethylsilyl)-1-propyne] (PTMSP) [6,7], and established by Swaidan et al. [8] as being due to a collapse in the larger micro-cavity elements within the morphology, creating a denser structure. This occurs because PIMs, being glassy polymers, are in a non-equilibrium state and hence, over time, slow polymer chain relaxation and motion alter the membrane’s morphology.

Several studies have investigated solutions to counter or reverse physical aging in PIMs-based membranes. The most common approach is a methanol wash, which removes residual casting solvent and permits the relaxation of polymer chains, leading to an increase in fractional free volume once the methanol is evaporated [9]. Similarly, chemically modifying or cross-linking PIMs-based membranes alter the permselectivity of the membrane and limit age-induced changes to the membrane’s performance [10,11,12]. Another approach has been to incorporate particles into the PIMs structure to form mixed matrix membranes, with evidence of reduced physical aging, dependent on the particle type [13,14]. Supercritical CO_2_ (scCO_2_) is an alternative treatment approach, which is known to alter the permselectivity of membranes because of the ability of scCO_2_ to solvate the polymer chains. This results in faster polymeric chain rearrangement, as well as the possibility of transitioning the polymer to a rubbery state and; therefore, plasticizes the membrane. Hence, morphology changes can occur on a shorter time frame under scCO_2_ conditions. In addition, scCO_2_ has the potential to swell the polymeric structure upon depressurization [15]. Hence, scCO_2_ has the potential to beneficially alter the performance of PIMs-based membranes for gas separation.

In this investigation, polymer of intrinsic microporosity (PIM-1)-based membranes were exposed to scCO_2_ for two different exposure time periods and two different depressurization rates. The resulting membranes gas separation performances were investigated in terms of helium, nitrogen, methane, oxygen and carbon dioxide permeabilities. In addition, the physical aging of the PIM-1 membranes after exposure to scCO_2_ was also measured, with the results analysed to evaluate the potential of scCO_2_ to alter PIM-1 gas separation and aging properties.

## 2. Materials and Methods

PIM-1, the polycondensation product of ultrahigh purity monomers of 5,5′,6,6′-tetrahydroxyl-3,3,3′,3′-tetramethyl-1,1′-spirobisindane (TTSBI) and 2,3,5,6-tetra fluoroterephthalonitrile (TFTPN), was synthesised following established procedures by Budd et al. [16]. Membranes of PIM-1 were cast from solutions of dichloromethane through controlled evaporation. The final film thickness was between 63 and 78 μm. All films were annealed at 150 °C for 1 day under vacuum to remove dichloromethane, and then cooled to room temperature overnight. The original membrane was washed with methanol and then allowed to dry to reverse any aging effects, while those membranes exposed to scCO_2_ were not [9,17]. Washing with methanol treatment of the scCO_2_ treated membranes reverts their morphology, similar to reversing aging effects.

Membrane densities were determined through standard procedures [18]. Gas sorption measurements of CO_2_ were undertaken on a gravimetric sorption analyser (GHP-FS, VTI Instruments) operating at 35 °C. The pressure was incrementally adjusted from 0 to 20 atm, with helium used for buoyancy correction [19]. Single gas permeabilities were undertaken on a variable pressure constant volume apparatus as previously described [20], with feed pressures of 8 atm and 35 °C. The permeability values were the average of three single gas measurements per gas with a fresh membrane each time; error margins corresponding to two standard deviations in the permeability data set. ScCO_2_ is achieved above 31.1 °C and 72.9 atm [21]. The scCO_2_ treatment was undertaken in an autoclave equipped with the inlet of CO_2_ and a backpressure regulator; membranes were exposed at 246.7 atm and at 50 °C for 2 or 8 h. Depressurization back to ambient pressure was achieved through two mechanisms, rapid depressurization, which occurred over a few minutes (depressurization rate: 118 atm/min), and gradual depressurization, over 150 min (depressurization rate: 1.7 atm/min). Hence, the scCO_2_ treatment process was designed to investigate both the impact of exposure time and removal rate of CO_2_ on the underlying PIM-1 membrane’s performance.

## 3. Results and Discussion

### 3.1. CO_2_ Sorption Isotherm of PIM-1

**Figure 1:**
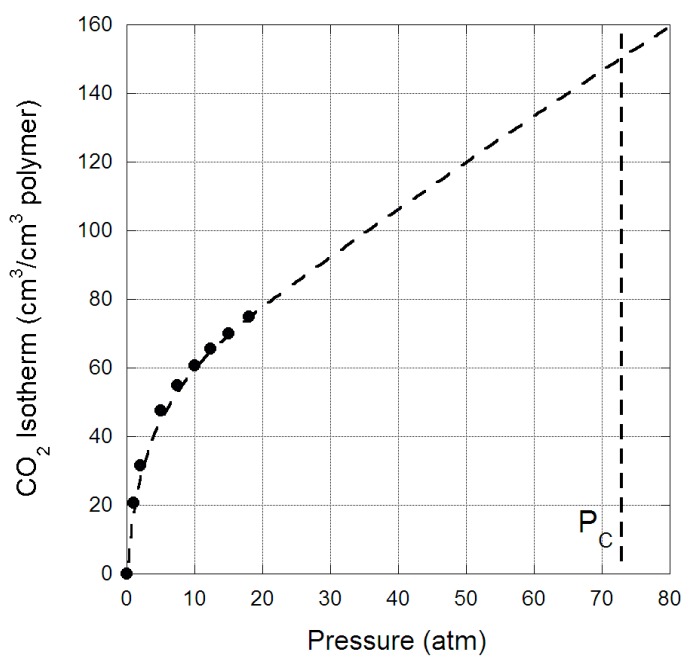
CO_2_ sorption isotherm (cm^3^/cm^3^ polymer) in original polymer of intrinsic microporosity (PIM-1) membrane as a function of pressure, the isotherm is extrapolated out to the critical pressure of CO_2_.

The sorption isotherm of CO_2_ in the original PIM-1 membrane is provided in Figure 1, as a function of pressure at 35 °C. This isotherm was comparable to literature and followed standard dual-sorption model behaviour [22], with a significant sorption of CO_2_ at low pressures attributed to the micro-cavities within the membrane morphology being filled, while at higher pressures the micro-cavities became saturated and additional sorption was limited to the polymeric matrix. The CO_2_ concentration (C) within the membrane can be modelled by dual-sorption theory [22]:

(1) $$C=k_{D}p+\frac{{C\prime }_{H}\mathrm{bp}}{\left(1+\mathrm{bp}\right)}$$

where p is the pressure, k_D_ the Henry’s law constant, C’_H_ the maximum Langmuir adsorption capacity and b the Langmuir affinity. The evaluated parameters are provided in Table 1.

These dual-sorption parameters were comparable to literature and reveal that the PIM-1 membrane studied here had similar morphology to those previous studies [10,23]. Interestingly, the k_D_ and b values for PIM-1 was significantly lower than other polymers investigated for CO_2_ separation, such as cellulose triacetate and polyimides [15]. This indicates that the PIM-1 polymer chain did not have strong affinity for CO_2_ relative to other polymers, and that the strong sorption was mainly attributed to the micro-cavities, which had a capacity significantly higher than other polymers.

The sorption analyser’s maximum pressure was 20 atm, and; therefore, determining the sorbed amount of CO_2_ up to the critical pressure could not be measured. However, the isotherm clearly followed standard dual-sorption mode behaviour, and hence it was possible to extrapolate the sorption behaviour to higher pressures to indicate how much CO_2_ could be sorbed into the membrane. This extrapolation is provided in Figure 1 only as a guide, since non-linear deviation in CO_2_ sorption is anticipated at significantly high pressures because of polymer plasticization and condensation of scCO_2_ in the membrane. Interestingly, the extrapolation suggests that PIM-1 membrane’s sorption of CO_2_ at high pressures was not substantial, only a doubling of the amount of CO_2_ sorbed at 20 atm. This is due to the relatively poor affinity the polymeric matrix has for CO_2_, compared to other polymers used for gas separation membranes [23]. Hence, the amount of CO_2_ sorbed at critical pressure would be comparable to cellulose triacetate [15], a midrange polymeric membrane with a permselectivity that is lower than the Robeson’s upper bound, rather than other high performing polymers.

### 3.2. Gas Permeability in Original PIM-1 Membrane

The gas permeability through the original PIM-1 membrane is provided in Table 2 after seven days of aging, along with reported literature values. There was discrepancy between the reported gas permeabilities in the literature, as well as with those determined here, which is attributed to the casting history of the PIM-1 membrane. The earlier study of Budd et al. [24] had lower gas permeabilities compared to this work and that of Thomas et al. [25], because the earlier studies had different degrees of physical aging and had not undergone methanol restoration. The CO_2_ permeability reported here had a reasonable correlation with that of Thomas et al. [25], though all gases were higher, especially CH_4_ which was three times the magnitude. However, the CH_4_ result was similar to the membrane reported by Starannikova et al. [26], and there was also comparison in the He and O_2_ permeabilities of the two membranes. Hence, the wide variation in reported gas permeabilities for PIM-1 based membranes could be attributed to the differences in the morphology of the membrane, as a result of both the synthesising procedure and casting history.

The selectivity of the original PIM-1 membrane is provided in Table 3, and displayed similar behaviour to literature, in that the membrane is clearly selective for CO_2_ against CH_4_ and N_2_; as well as being selective for O_2_ and He. However, the high CH_4_ permeability of this membrane resulted in the CO_2_/CH_4_ and He/CH_4_ selectivity being lower than literature, and hence the membrane investigated here was not on the Robeson’s upper bound for these gas pairs.

### 3.3. Supercritical CO_2_ Treatment

#### 3.3.1. Density

**Figure 2:**
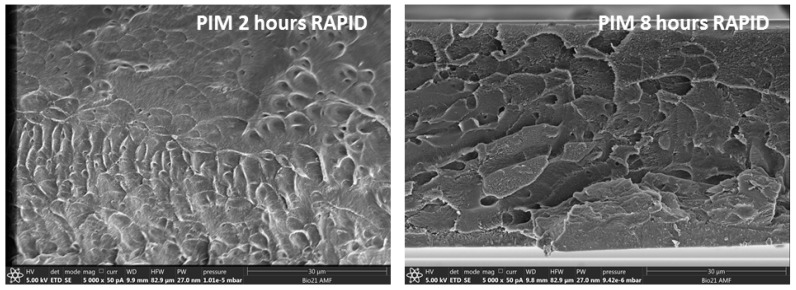
Scanning electron microscope (SEM) images of the PIM-1 membranes after two- and eight-h exposure to scCO_2_, with rapid depressurization.

The density of the original PIM-1 membrane and after treatment with scCO_2_ for two and eight hours, as well as rapid and slow depressurizations, is provided in Table 4, after seven days of aging. The density of the original membrane, after methanol regeneration, was 1.114 g/cm^3^. After scCO_2_ treatment, the density increased irrespective of the treatment protocol, and hence a denser morphology was obtained. This was associated with the ability of scCO_2_ to solubilise the polymer, enabling enhanced chain mobilization and rearrangement. As a consequence, the fractional free volume of scCO_2_-treated PIM-1 membrane would have reduced. Importantly, the longer eight-hour exposure resulted in a denser membrane morphology, than the shorter two-hour exposure, supporting the conclusion that polymer solubilisation was the dominate factor. The depressurization rate clearly impacted the morphology, with the rapid depressurization resulting in a lower density structure, irrespective of exposure time, which has been observed for other polymeric membranes exposed to scCO_2_ [15]. This dense morphology was clearly observed in SEM images for the rapid depressurization PIM-1 membranes, provided in Figure 2. The densities were greater than the original membrane, implying that the morphology changes due to rapid depressurization of the scCO_2_ were not great enough to reverse the polymer solubilisation effect. This density increase within the PIM-1 membranes differed from that observed for cellulose triacetate membranes exposed to scCO_2_, irrespective of depressurization, as well as polyimide-based membranes that underwent rapid depressurization, but was similar to polyimide membranes that experienced slow depressurization [15]. Hence, scCO_2_ treatment was polymer dependent.

#### 3.3.2. Gas Permeability

The gas permeability through the PIM-1 membrane after treatment with scCO_2_ is provided in Table 4, after seven days of aging, for two and eight-hour exposure, as well as rapid and slow depressurization. All four scCO_2_ treatments resulted in a reduction in the gas permeability through the membrane, compared to the original membrane (Table 2). This behaviour corresponded well with the increased density of the scCO_2_ treated membranes, implying that the PIM-1 morphology change restricts gas permeance. The exception was N_2_, which was essentially unchanged, within error, of the original membrane result. The low permeability of N_2_ in the original and scCO_2_ treated membranes implies that the PIM-1 membrane morphology remained unfavourable for N_2_, and the interaction with scCO_2_ did not alter this.

There was a clear trend in the scCO_2_ treatment protocol on the membrane, with rapid depressurization having reduced gas permeabilities compared to slow depressurization, for the same exposure time. This reveals that rapidly removing the scCO_2_ from the PIM-1 membrane alters the morphology to be less favourable for gas permeance compared to slow depressurization. This was counter to the observation for other polymeric membranes exposed to scCO_2_, notably cellulose triacetate and polyimide [15], where rapid depressurization resulted in swelling. This behaviour was also counter to the density measurements (Table 4), where a lower density usually corresponded to increased gas permeability. This difference was attributed to the higher fractional free volume of PIM-1 and the already high permeance of CO_2_ enabling the scCO_2_ to rapidly desorb through the established connecting pathways between micro-cavities. This limited the ability of scCO_2_ to generate new pathways for desorption during rapid depressurization and, hence, the PIM-1 structure did not swell, as the resulting densities of the rapid depressurization membranes remained greater than the original membrane. Why the slower depressurization protocol resulted in higher gas permeabilities is unknown, given the denser morphology (Table 4); but the behaviour does suggest that the existing and established pathways through PIM-1 membranes’ micro-cavities remained open during scCO_2_ treatment, most likely because of the strong accumulation of scCO_2_ in these free volume regions. The variability in gas permeability after scCO_2_ treatment of various polymeric membranes [15] further establishes that the change in morphology and gas separation properties outcomes are polymer dependent.

The corresponding selectivity of the PIM-1 membranes after scCO_2_ treatment are provided in Table 5. Compared to the original membrane there was clear deviation for the scCO_2_ membranes. For separation from CH_4_ (i.e., CO_2_/CH_4_ and He/CH_4_) the scCO_2_-treated membranes showed an increase in selectivity over the original membrane. This was a direct result of the CH_4_ permeability reducing by 32%–39% after exposure to scCO_2_, while the CO_2_ permeability was reduced by only 7%–32% and He permeability reduced by 4%–31%. In contrast, CO_2_/N_2_ and He/N_2_ selectivity decreased after exposure to scCO_2_, which was a direct result of the N_2_ permeability, through the scCO_2_-treated PIM-1 membranes, remaining essentially constant with the original membrane. Furthermore, there was evidence that longer scCO_2_ exposure time and slower depressurization result in higher selectivity than rapid depressurization.

#### 3.3.3. Aging Study

**Figure 3:**
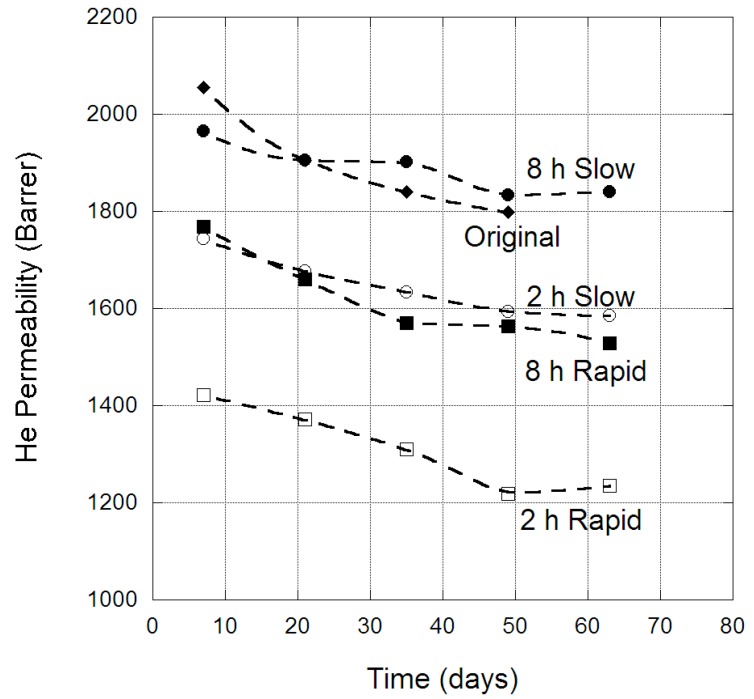
He permeability (barrer) in PIM-1 membranes over time, for the original and scCO_2_-treated states, at 35 °C.

**Figure 4:**
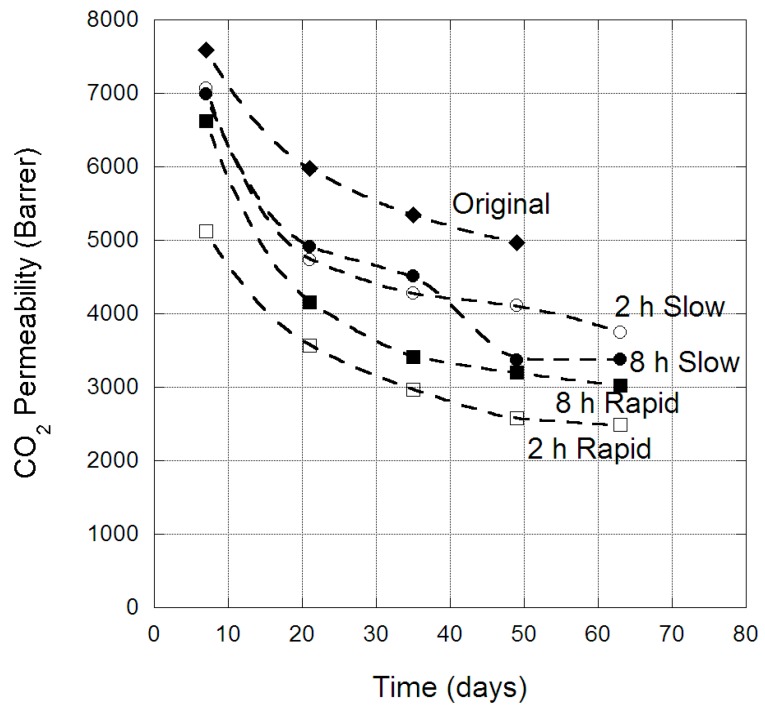
CO_2_ permeability (barrer) in PIM-1 membrane over time, for the original and scCO_2_-treated states, at 35 °C.

**Figure 5:**
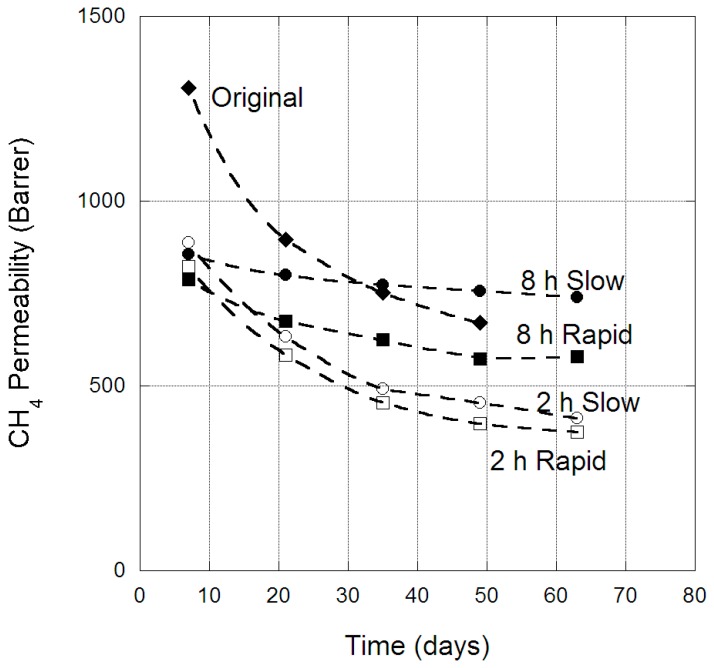
CH_4_ permeability (barrer) in PIM-1 membrane over time, for the original and scCO_2_-treated states, at 35 °C.

**Figure 6:**
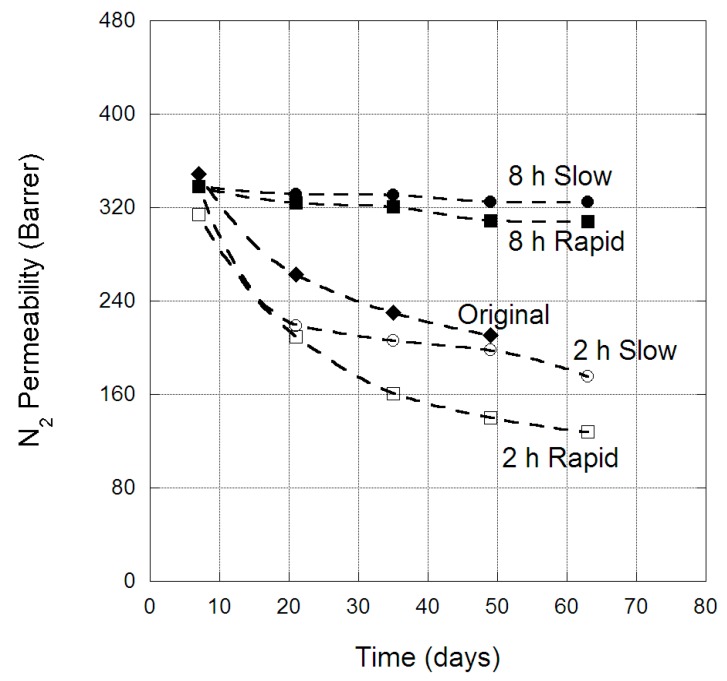
N_2_ permeability (barrer) in PIM-1 membrane over time, for the original and scCO_2_-treated states, at 35 °C.

**Figure 7:**
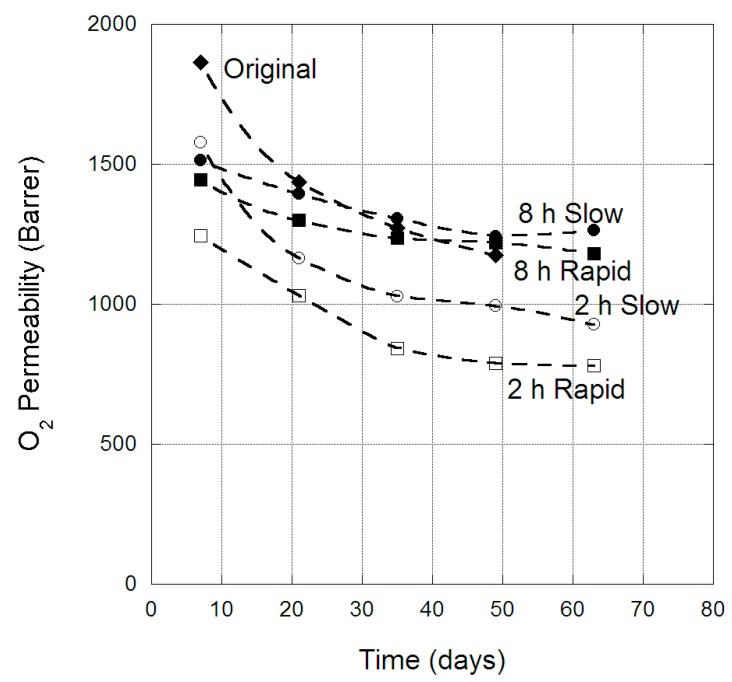
O_2_ permeability (barrer) in PIM-1 membrane over time, for the original and scCO_2_-treated states, at 35 °C.

The change in He permeability through PIM-1 membranes over an extended period of time is provided in Figure 3, for both the original membrane and the four scCO_2_-treated protocols. For all five membranes the He permeability decreased with time, indicative of physical aging. There were differences between the permeabilities presented in Figure 3, Figure 4, Figure 5, Figure 6 and Figure 7, and those presented in Table 4, as they represent different PIM-1 membranes that were measured at different times after scCO_2_ treatment. Over the 63 days studied, all five membranes experienced similar aging behaviour, losing ~200 barrer in He permeability at the 63-day mark. Interestingly, there was no difference between the original and scCO_2_-treated membranes in terms of He aging. This suggests scCO_2_ exposure had not changed the mechanism of physical aging; that of lattice contraction within the PIM-1 structure [8], as polymer chains rearrange to a denser state. A comparable increase in density of the PIM-1 membranes was observed in the aging period, with the two hours slow depressurization PIM-1 membrane experiencing a density increase of 5%. However, He was not a good indicator of micro-cavity change, because being the smallest molecule enabled He to permeate more readily through the micro-cavities and polymeric matrix compared to other gases.

The change in CO_2_ permeability through PIM-1 membranes over an extended period of time is provided in Figure 4, for both the original membrane and the four scCO_2_-treated protocols. Again, there was clear evidence of physical aging, with an initial pronounced loss in permeability over the first two weeks, which progressed to a gradual reduction over the longer time period. The effect of physical aging on He and CO_2_ permeabilities was clearly different, which is attributed to the relative sizes of the gases. CO_2_ is significantly large that its permeance through the PIM-1 morphology is dominated by transport through the micro-cavities, which becomes restricted as the membrane ages. The original membrane CO_2_ permeability was reduced by ~2500 barrer over the 49 days studied, while the scCO_2_-treated membranes experienced a larger reduction of 2500 to 3500 barrer over the aging period. Hence, the scCO_2_ treated process had enhanced the physical aging impact on CO_2_ permeability. This is attributed to the denser membrane structure (Table 4), resulting from scCO_2_ treatment having reduced the micro-cavities in which CO_2_ transverses through the PIM-1 membrane, and hence physical aging in the remaining micro-cavities was more pronounced on CO_2_ permeability, which appeared as an enhancement of physical aging.

The change in CH_4_ permeability through PIM-1 membranes over time is provided in Figure 5, for both the original membrane and the four scCO_2_-treated protocols. For CH_4_, there was a clear difference in the physical aging behaviour of the original membrane and those treated with scCO_2_. The original membrane CH_4_ permeability was reduced by ~500 barrer over 50 days, while membranes exposed to scCO_2_ for two hours experienced a physical aging loss of ~300 barrer, and membranes treated for eight hours experienced less than 200 barrer loss in CH_4_ permeability over 63 days. Similar behaviour was also clearly observed for N_2_ and O_2_ permeability in PIM-1 membranes over time, as provided in Figure 6 and Figure 7, respectively. Hence, exposure to eight hours of scCO_2_ resulted in a morphology that underwent minor physical aging in terms of CH_4_, N_2_ and O_2_ permeability, while two hours scCO_2_ exposure gave rise to physical aging that was significantly reduced compared to the original membrane for the same gases. This was attributed to the scCO_2_ treatment reducing the larger micro-cavities, by creating a denser morphology, in which CH_4_, N_2_ and O_2_ would previously permeate through PIM-1 (as evident by the decrease in permeability of CH_4_ and O_2_ between the original and scCO_2_ treated membranes). Hence, further micro-cavity contraction due to physical aging had only a minor impact on CH_4_, N_2_ and O_2_, as they were already size excluded from the micro-cavities in which CO_2_ and He permeate. This became notable in the change in selectivity of the membrane over time, with the CO_2_/N_2_ selectivity of the eight-hour exposed membrane (irrespective of depressurization protocol) decreasing to 10.3 over the 63 days. Similar, changes were also observed in the CO_2_/CH_4_ selectivity, which decreased to 4.6 over the 63 days; highlighting the magnitude of the decrease in CO_2_ permeability relative to the larger gases.

**Figure 8:**
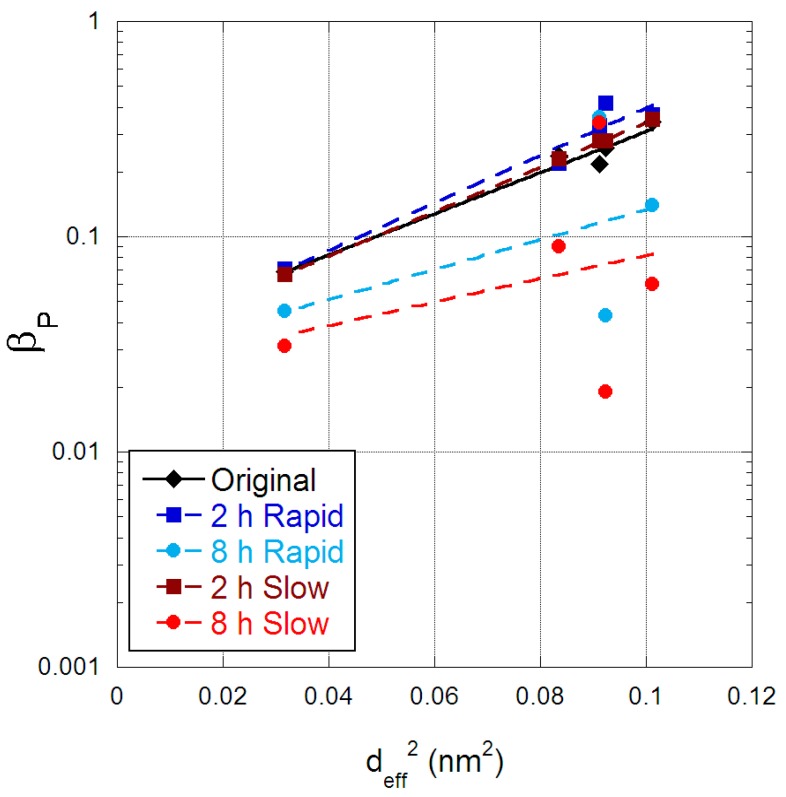
Aging rate constant for PIM-1 membranes as a function of the square of the effective gas diameter (nm^2^).

The physical aging of gas permeability in PIM-1 membranes can be described by a power law, as established by Bernardo et al. [27]:

(2) $$P=P_{0}t^{-\beta_{P}}$$

where P_0_ is the initial permeability of the membrane at t = 1 and β_P_ the permeability aging rate constant. The determined β_P_ values for the original and scCO_2_-treated PIM-1 membranes are provided in Figure 8, as a function of the squared effective diameter of the gases studied, CO_2_ is at 0.091 nm^2^ and N_2_ is at 0.092 nm^2^. The original PIM-1 membrane had very similar β_P_ values to that reported by Bernardo et al. [27], and hence comparable physical aging with that study, which included an ethanol treatment post-fabrication. For the scCO_2_-treated PIM-1 there was a clear reduction in the aging constant for those membranes exposed for eight hours, along with a loss in correlation associated with gas diameter. The explanation to this behaviour is attributed to CO_2_ solubilising the polymer chain and creating a denser morphology the longer PIM-1 is exposed; however, scCO_2_ when desorbing leaves behind micro-cavities of sufficient size to enable depressurization. These micro-cavities then undergo physical aging, which was observed in the permeability loss of He and CO_2_; while CH_4_, N_2_ and O_2_ are larger molecules and thus size restricted from these post scCO_2_ micro-cavities, and hence do not experience physical aging to the same degree as their permeabilities are already reduced.

## 4. Conclusions

The permeability and selectivity of PIM-1 membranes for gas separation are impacted by exposure to scCO_2_, due to the process creating a denser membrane morphology. He, CH_4_, O_2_ and CO_2_ all experience a reduction in permeability through the membrane, while N_2_ permeability remains relatively constant, when PIM-1 is exposed to scCO_2_ for two or eight hours, at the end of which the process undergoes rapid or slow depressurization. This is attributed to scCO_2_ solubilising the PIM-1 polymer chain, enabling polymer chain rearrangement and altering the micro-cavity environment. Interestingly, the physical aging of PIM-1 membranes is altered by exposure to scCO_2_. He and CO_2_ permeabilities are observed to decrease significantly over extend time periods due to contraction of the micro-cavities within PIM-1; however, N_2_, CH_4_ and O_2_ permeabilities experience only a small reduction over the same time period, especially if the PIM-1 membrane had been exposed to scCO_2_ for eight hours. This is attributed to the initial scCO_2_ exposure contracting the larger micro-cavities, reducing N_2_, CH_4_ and O_2_ permeabilities because of size exclusion, as such subsequent aging in those micro-cavities does not impact those gases permeabilities because they are already excluded. Hence, scCO_2_ exposure is a procedure that has potential implications in reducing the impact of physical aging in PIM-1-based membranes for N_2_, CH_4_ and O_2_ separation.

## Figures and Tables

**Table 1:** Dual-sorption parameters (Henry’s law constant (k_D_), maximum Langmuir capacity (C’_H_) and Langmuir affinity (b) for CO_2_ in PIM-1 membrane at 35 °C.

Parameter	This Work	Mason et al. [10]
k_D_ (cm^3^/cm^3^ atm)	1.3 ± 0.1	3.86
C’_H_ (cm^3^/cm^3^)	56 ± 2	52.8
b (atm^−1^)	0.52 ± 0.03	0.706

**Table 2:** Gas permeability (barrer) in the original PIM-1 membrane at 35 °C, along with literature values.

	This Work	Budd et al. [24]	Staiger et al. [5]	Thomas et al. [25]	Starannikova et al. [26]
He	2055 ± 90	660	1061	1500	1740
N_2_	349 ± 8	92	238	340	830
O_2_	1865 ± 75	370	786	1300	2390
CH_4_	1307 ± 26	125	360	430	1440
CO_2_	7595 ± 84	2300	3496	6500	15,300

**Table 3:** Selectivity of PIM-1 membrane at 35 °C.

	This Work	Budd et al. [24]	Staiger et al. [5]	Thomas et al. [25]	Starannikova et al. [26]
CO_2_/CH_4_	5.8 ± 0.2	18.4	9.7	15.1	10.6
CO_2_/N_2_	21.8 ± 0.7	25.0	14.7	19.1	18.4
O_2_/N_2_	5.3 ± 0.3	4.0	3.3	3.8	2.9
He/N_2_	5.9 ± 0.4	7.2	4.5	4.4	2.1
He/CH_4_	1.6 ± 0.1	5.3	2.9	3.5	1.2

**Table 4:** Density (g/cm^3^) and gas permeability (barrer) in supercritical CO_2_-treated PIM-1 membrane at 35 °C. The original untreated PIM-1 membrane had a density of 1.114 g/cm^3^.

Exposure Time	2 h		8 h	
Depressurization	Rapid	Slow	Rapid	Slow
Density (g/cm^3^)	1.142	1.215	1.184	1.306
Permeability (barrer)				
He	1421 ± 88	1768 ± 92	1744 ± 68	1966 ± 72
N_2_	314 ± 19	338 ± 12	338 ± 14	338 ± 16
O_2_	1245 ± 64	1577 ± 69	1444 ± 63	1513 ± 72
CH_4_	823 ± 21	887 ± 24	788 ± 16	857 ± 22
CO_2_	5118 ± 157	7068 ± 83	6621 ± 164	6989 ± 96

**Table 5:** Selectivity of supercritical CO_2_-treated PIM-1 membrane at 35 °C.

Exposure Time	2 h		8 h	
Depressurization	Rapid	Slow	Rapid	Slow
CO_2_/CH_4_	6.2 ± 0.3	8.0 ± 0.3	8.4 ± 0.4	8.2 ± 0.3
CO_2_/N_2_	16.3 ± 1.5	20.9 ± 1.0	19.6 ± 1.3	20.7 ± 1.3
O_2_/N_2_	4.0 ± 0.4	4.7 ± 0.4	4.3 ± 0.4	4.5 ± 0.4
He/N_2_	4.5 ± 0.6	5.2 ± 0.5	5.2 ± 0.4	5.8 ± 0.5
He/CH_4_	1.7 ± 0.1	2.0 ± 0.2	2.2 ± 0.1	2.3 ± 0.1
